# Physiological regulation of phosphate by vitamin D, parathyroid hormone (PTH) and phosphate (Pi)

**DOI:** 10.1007/s00424-018-2231-z

**Published:** 2018-11-05

**Authors:** Grégory Jacquillet, Robert J. Unwin

**Affiliations:** 1 Centre for Nephrology, University College London (UCL), Royal Free Campus, Rowland Hill Street, London, NW3 2PF UK; 2 AstraZeneca IMED ECD CVRM R&D, Gothenburg, Sweden

**Keywords:** Phosphate, Homeostasis, Renal, Kidney physiology, Epithelial transport, Proximal tubule

## Abstract

Inorganic phosphate (Pi) is an abundant element in the body and is essential for a wide variety of key biological processes. It plays an essential role in cellular energy metabolism and cell signalling, e.g. adenosine and guanosine triphosphates (ATP, GTP), and in the composition of phospholipid membranes and bone, and is an integral part of DNA and RNA. It is an important buffer in blood and urine and contributes to normal acid-base balance. Given its widespread role in almost every molecular and cellular function, changes in serum Pi levels and balance can have important and untoward effects. Pi homoeostasis is maintained by a counterbalance between dietary Pi absorption by the gut, mobilisation from bone and renal excretion. Approximately 85% of total body Pi is present in bone and only 1% is present as free Pi in extracellular fluids. In humans, extracellular concentrations of inorganic Pi vary between 0.8 and 1.2 mM, and in plasma or serum Pi exists in both its monovalent and divalent forms (H_2_PO_4_^−^ and HPO_4_^2−^). In the intestine, approximately 30% of Pi absorption is vitamin D regulated and dependent. To help maintain Pi balance, reabsorption of filtered Pi along the renal proximal tubule (PT) is via the NaPi-IIa and NaPi-IIc Na^+^-coupled Pi cotransporters, with a smaller contribution from the PiT-2 transporters. Endocrine factors, including, vitamin D and parathyroid hormone (PTH), as well as newer factors such as fibroblast growth factor (FGF)-23 and its coreceptor α-klotho, are intimately involved in the control of Pi homeostasis. A tight regulation of Pi is critical, since hyperphosphataemia is associated with increased cardiovascular morbidity in chronic kidney disease (CKD) and hypophosphataemia with rickets and growth retardation. This short review considers the control of Pi balance by vitamin D, PTH and Pi itself, with an emphasis on the insights gained from human genetic disorders and genetically modified mouse models.

## Renal Pi transporters

In the early 1990s, a Na^+^-coupled Pi cotransport system (NaPi-1) was first identified in the rabbit kidney cortex from expression cloning using *Xenopus laevis* oocytes and tracer flux studies with ^32^P-inorganic Pi [17]. However, due to a lack of any response of NaPi-1 expression in oocytes to physiological changes in Pi concentrations, the authors concluded that NaPi-1 did not match the physiological function and characteristics determined from isolated renal brush border membrane (BBM) vesicle studies of renal Na^+^-coupled Pi cotransport [165]. However, soon after this, additional Na^+^-coupled Pi-cotransporters were identified in rat and human kidney cortex (NaPi-2 and NaPi-3) [88]. NaPi-2-related mRNA and protein are expressed in the BBM of the proximal tubule (PT) and their abundance varies with changes in dietary Pi intake, as well as changes in serum parathyroid hormone (PTH) levels [88].

The weak overall homology between NaPi-1 and NaPi-2/3 led to classification of the Na/Pi-cotransport system into two groups: type I (NaPi-1-related) and type II (NaPi-2-related) Na^+^-Pi-cotransporters [88]; a third group of Na^+^-coupled Pi cotransporter proteins was described in 1996. First identified as a retroviral receptor for Gibbon Ape leukaemia virus (Glvr-1; [105, 153]) and for rat amphotropic virus (Ram-1; [91, 166]), it was shown that when expressed in Xenopus oocytes, these membrane receptor proteins exhibited Pi transport activity in a Na^+^-dependent manner [71]. Considering their ability to transport Pi, these proteins were then renamed PiT-1 and PiT-2, respectively [70]. cDNA sequences related to PiT-1 and PiT-2 have been cloned from human [105, 153], rat [90] and Chinese hamster [166] tissues.

The Na^+^-coupled Pi cotransporter proteins have now been classified into two groups: the SLC34 family (type II Na^+^-coupled Pi transporters: NaPi-IIa, NaPi-IIb, NaPi-IIc) and the SLC20 family (Type III Na^+^-coupled Pi cotransporters, PiT-1, PiT-2). In the proximal tubule (PT), at least three different Na^+^-coupled Pi cotransporters mediate the initial step of Pi reabsorption across the apical BBM: NaPi-IIa (SLC34A1), NaPi-IIc (SLC34A3), and PiT-2 (SLC20A2) [96].

## The SLC34 family of Na^+^-coupled pi cotransporters

### NaPi-IIa (SLC34A1)

In humans, specific mRNA expression of NaPi-IIa has been detected exclusively in the kidney [104]. The major site of expression of NaPi-IIa occurs mainly in the renal PT at the apical BBM as an 80 to 90 kDa protein (639 amino acids) [36]. Under normal conditions, the abundance of NaPi-IIa is highest in the S1 segment of the PT of juxtamedullary nephrons [36], whereas during Pi depletion, expression is also observed in the S2 and S3 segments [81]. Loss-of-function mutations in NaPi-IIa have been associated with hypophosphataemia, kidney stones, nephrocalcinosis, the renal Fanconi syndrome, and chronic kidney disease [89, 120].

### NaPi-IIb (SLC34A2)

In humans, NaPi-IIb mRNA has been detected in lung, testis, salivary gland, thyroid gland, small intestine, liver, mammary gland and uterus, but not in renal tissue [104]. However, a recent study in rat kidney has reported detection of NaPi-IIb expression by in situ hybridisation in the distal nephron, with a seemingly paradoxical increase in expression on a high Pi diet, which is hypothesised to reflect an adaptive and potentially secretory role in this location [140].

### NaPi-IIc (SLC34A3)

In humans, the distribution of NaPi-IIc is kidney specific [104]. Like NaPi-IIa, the localisation of NaPi-IIc as a 75-kD protein (599 amino acids) is at the apical BBM of the PT of juxtamedullary nephrons [122]. Mutations in SLC34A3 can cause hypophosphataemic rickets, hypercalciuria, increased 1,25-(OH)2D3, nephrocalcinosis and nephrolithiasis [16, 61, 87].

## The SLC20 family of Na^+^-coupled Pi cotransporters

### PiT-1 and PiT-2

The mRNAs for the isoforms of the two members of the SLC20 family, PiT-1 and PiT-2, have been described as ubiquitously expressed in rodent and human tissues [31, 104]. PiT-2 is localised to the BBM of the PT, colocalising with NaPi-IIa and NaPi-IIc [112, 156]. The expression of both PiT-1 and PiT-2 has also been reported in the intestine [10, 50]. However, no renal phenotype or disorder related to mutations in SLC20 Pi transporters have been reported to date. In the kidney, the role of SLC20A2 (PiT-2) in renal Pi handling and inherited disorders of mineral balance remains unclear.

## Renal Pi transport: kinetics and structure-function

In the kidney, Pi reabsorption occurs primarily via a transcellular pathway in the PT, paracellular reabsorption being considered insignificant [68]. Na^+^-coupled Pi cotransporters NaPi-IIa, NaPi-IIc and PiT-2 are the three transporters identified to be responsible for the apical uptake of Pi from the glomerular filtrate [96]. Apical Pi entry is facilitated by active inward flux of Na^+^, which is matched by the action of the basolateral Na^+^/K^+^-ATPase. All three isoforms of the SCL34 family show a preference for divalent Pi (HPO_4_^2−^). pH is another regulator of Pi transport in the PT: protons (H^+^) can directly modulate the Pi transporter or indirectly by changing the monovalent/divalent Pi ratio in the lumen of the PT [46]. NaPi-IIa is electrogenic (couples 3 Na^+^ to 1 Pi), whereas NaPi-IIc is electroneutral (couples 2 Na^+^ to 1 Pi) [6, 122]. Animal studies have demonstrated that NaPi-IIa is the major renal Pi transporter, because NaPi-IIa-deficient mice exhibit severe hypophosphataemia due to urinary Pi losses [14]. In contrast, NaPi-IIc deficiency is associated with normal serum Pi and urinary Pi excretion [124]. However, in humans, it seems that NaPi-IIc plays a more important role in calcium homeostasis (see later). The capacity of Pi transport in the PT is mainly determined by the abundance of Na^+^-coupled Pi cotransporters, which is controlled by multiple factors that can regulate Pi homeostasis, including dietary Pi intake, PTH, 1,25(OH)2D3 and FGF-23/klotho.

The 3-D structure of mammalian SCL34 is unknown, but it is supposed that SLC34 proteins comprise 12 transmembrane-spanning domains of which the intracellular linker region is critical for PTH sensitivity [44, 67].

Na^+^-coupled Pi cotransport mediated by PiT-2, which is electrogenic (couples 2 Na^+^ to 1 Pi), has a greater affinity for monovalent Pi ions (H_2_PO_4_^−^) [116, 118]. In contrast with SLC34 proteins, SCL20 Pi transport is insensitive to a reduced pH and is not only exclusively driven by Na^+^ but also by Li^+^ [155]. In the absence of Na^+^, a drop in pH from 7.5 to 6.0 allows PiT-2 to transport Pi, suggesting that Na^+^ can also be replaced by H^+^ for this transporter [21]. As for SLC34 proteins, the proposed topology for SLC20 proteins is a 12 transmembrane domain protein; however, relatively few structure-function studies have been undertaken. The contribution of PiT-2 to Pi uptake in the PT is still unclear, but it has been assumed to be small, accounting for approximately 5% of the total renal Pi reabsorption [157]. In the kidney, few metabolic factors participate in the regulation of PiT-2 expression, including dietary Pi, K^+^ deficiency or metabolic acidosis [24, 157].

To date, the basolateral exit pathway for Pi remains unknown, although a candidate has emerged recently [54]. Nephron-specific knockout of the xenotropic and polytropic retroviral receptor gene *XPR1* in mice resulted in hypophosphataemia and hyperphosphaturia, suggesting a role for this transporter in renal tubular Pi reabsorption. This protein is of some interest, because of its high degree of homology with PHO1, a Pi extrusion transporter found in plants, which has been shown to mediate Pi transport from roots to shoots [54]. XPR1 has also been shown to mediate Pi efflux in cells in vitro [49]; however, more studies are needed to clarify the exact role of XPR1 in basolateral Pi extrusion.

## Hormonal factors that affect Pi balance

### Vitamin D

In the kidney, the PT is the major site of 1,25(OH)2D3 synthesis, as well as the main site of Pi absorption. Under normal dietary conditions, ~ 97% of filtered Pi is reabsorbed across the apical BBM by Na^+^-coupled Pi cotransporters NaPi-IIa and NaPi-IIc [157]. This reabsorption can be regulated by several factors, including FGF23, PTH and 1,25(OH)2D3 itself. PTH and FGF23 promote renal Pi loss by stimulating the internalisation and lysosomal degradation of the transporters [9] or by decreasing their expression [170]. In contrast, 1,25(OH)2D3 stimulates Pi absorption by decreasing the PTH level [15].

**Fig. 1:**
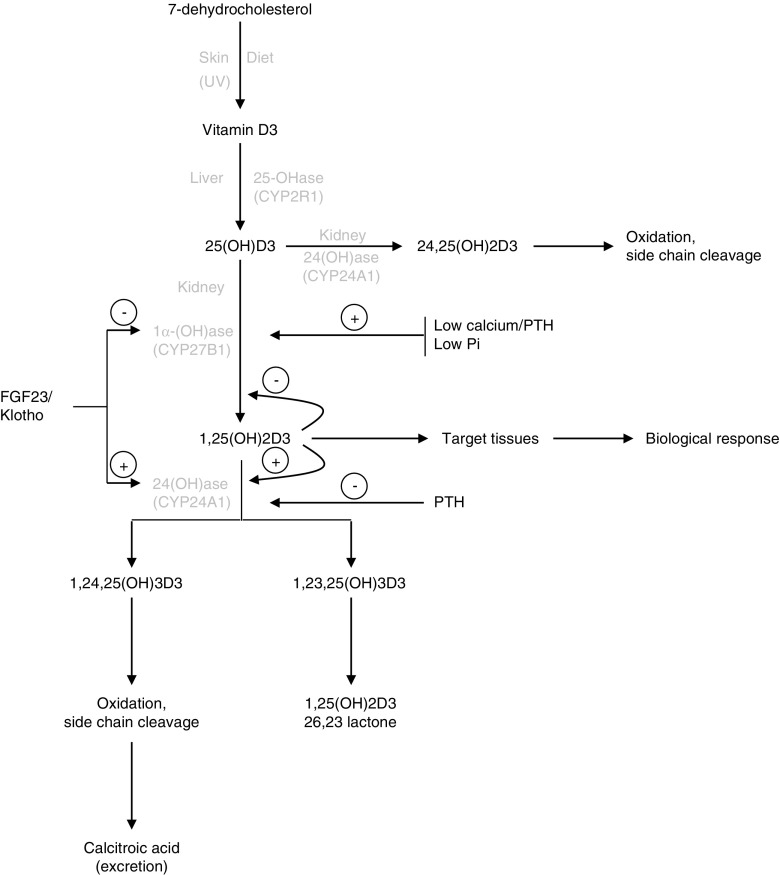
The metabolic pathway for vitamin D (Adapted/modified from Schlingmann et al. [119].)

Vitamin D3 (cholecalciferol), the natural form of vitamin D, is a steroid hormone that can be synthesised endogenously or taken in from the diet (see Fig. 1). In the skin, irradiation of 7-dehydrocholesterol produces pre-vitamin D3 that is immediately converted to vitamin D3 (cholecalciferol). The production of vitamin D in the skin is the most important source of vitamin D and depends on the intensity of UV irradiation and exposure. Vitamin D can also be provided to a small extent in the diet, being mainly present in fish oils and fortified dairy products [52].

In its native form, vitamin D3 is not biologically active: as a precursor of vitamin D, it is transported to the liver by vitamin D-binding protein (DBP) and in the liver hydroxylated by a 25-hydoxylase enzyme (25-OHase) to produce 25-hydroxyvitamin D3 (25(OH)D3). Studies conducted in humans have shown that CYP2R1 is a good candidate for the enzymatic conversion of vitamin D3 to 25(OH)D3, since patients with a mutation in CYP2R1 have a deficiency of 25(OH)D3 and exhibit signs and symptoms of vitamin D deficiency and develop rickets [147, 148]. In animals, CYP2R1 has also been shown to be the main enzyme responsible for 25-hydroxylation of vitamin D [172]. 25(OH)D3 concentration, the major circulating form of vitamin D measured in blood, is used by clinicians as an index of vitamin D status [55]. It has not been shown that synthesis of 25(OH)D3 is highly regulated, but the production of 25(OH)D3 in *Cyp2r1* null mice, although very low, is not completely abolished, suggesting the presence of other 25-hydroxylases not yet identified [172].

25(OH)D3 is a biologically inactive form of vitamin D and needs to be hydroxylated to 1,25(OH)2D3, the active form of vitamin D. Bound to DBP, 25(OH)D3 is then transported to the kidney where it is filtered by the glomerulus and taken up by the PT via endocytic internalisation involving the megalin/cubilin surface receptor system [30]. Megalin is thought to be the key protein for renal DBP/25(OH)D3 uptake and a major component in vitamin D synthesis, since megalin knockout mice show a phenotype similar to that observed in vitamin D-deficient rickets [80]. Hydroxylation of 25(OH)D3 to form 1,25(OH)2D3, the hormonally active and functional form of vitamin D, occurs predominantly in the kidney proximal straight tubule (S3) [66]. The enzyme responsible for the conversion of 25(OH)D3 to 1,25(OH)2D3 is renal 25(OH)D3 1-α-hydroxylase (mitochondrial CYP27B1), which largely determines the circulating concentrations of 1,25(OH)2D3 [130, 136, 142]. The importance of CYP27B1 has been confirmed in *Cyp27b1* null mice (see later). The presence of extrarenal expression of CYP27B1 has also been shown in the macrophages of patients with sarcoidosis and Crohn’s disease, in some cancer cells and in the parathyroid gland [1, 41, 171]; all clinical settings in which hypervitaminosis D and hypercalcaemia can occur. However, whether there is a functionally important impact of CYP27B1 activity in vivo at sites other than the kidney and placenta under normal physiological conditions is unclear.

#### Regulation of 1,25(OH)2D3 production

Vitamin D synthesis, and thereby renal Pi reabsorption, can also be regulated by a direct action on the enzymes responsible for vitamin D synthesis. CYP27B1 expression is upregulated by PTH but downregulated by FGF23 and 1,25(OH)2D3 [85, 173]. Besides the action of PTH and FGF23 on 1,25(OH)2D3 formation, hydroxylation of 1,25(OH)2D3 and its precursors can also regulate the amount of vitamin D produced by the kidney and thereby regulate Pi loss; 25(OH)D3 can be converted to 24,25(OH)2D3 by hydroxylation with CYP24A1, a mitochondrial inner-membrane cytochrome P-450 enzyme [65]. This enzyme can hydroxylate both 25(OH)D3 and 1,25(OH)2D3, the latter being considered the preferred substrate for CYP24A1 [129]. By catalysing the conversion of 1,25(OH)2D3 to 24,25(OH)2D3 or 1,25(OH)2D3-26,23 lactone, which is rapidly excreted, CYP24A1 limits the circulating concentrations of 1,25(OH)2D3 [65, 129]. CYP24A1 is also known to convert 25(OH)D3 to 24,25(OH)D3 or 25(OH)D3-26,23 lactone, reducing the synthesis of active 1,25(OH)2D3 [65]. Although 1,25(OH)2D3 can regulate its own production by inhibiting CYP27B1 [23], further studies are needed to determine genome-wide mechanisms involved in 1,25(OH)2D3-mediated suppression of CYP27B1. When compared with the regulation of CYP27B1, CYP24A1 is reciprocally regulated: stimulated by 1,25(OH)2D3 and inhibited by low calcium and PTH [18, 56, 113].

In addition to upregulation by PTH and downregulation by increased serum calcium and Pi levels, 1,25(OH)2D3 concentration is also downregulated by increased FGF23 levels. FGF23 is an important physiological regulator of vitamin D metabolism that results in renal Pi excretion by decreasing reabsorption in the PT [170]. In parallel, α-klotho, a transmembrane protein that is highly expressed in the renal distal tubule (DT), acts as an obligate coreceptor for FGF23; α-klotho is also expressed at lower levels in the PT. Together, FGF23 and α-klotho, by suppressing the expression of CYP27B1 and inducing CYP24A1, can inhibit the synthesis and promote the catabolism of 1,25(OH)2D3 [60]. Indeed, the phenotypes of FGF23 and α-klotho deficiency are very similar, with hyperphosphataemia and increased synthesis of 1,25(OH)2D3, and indicate the cooperative action of α-klotho and FGF23 in a common signalling pathway [79, 128].

#### Effect of vitamin D on the parathyroid gland

**Fig. 2:**
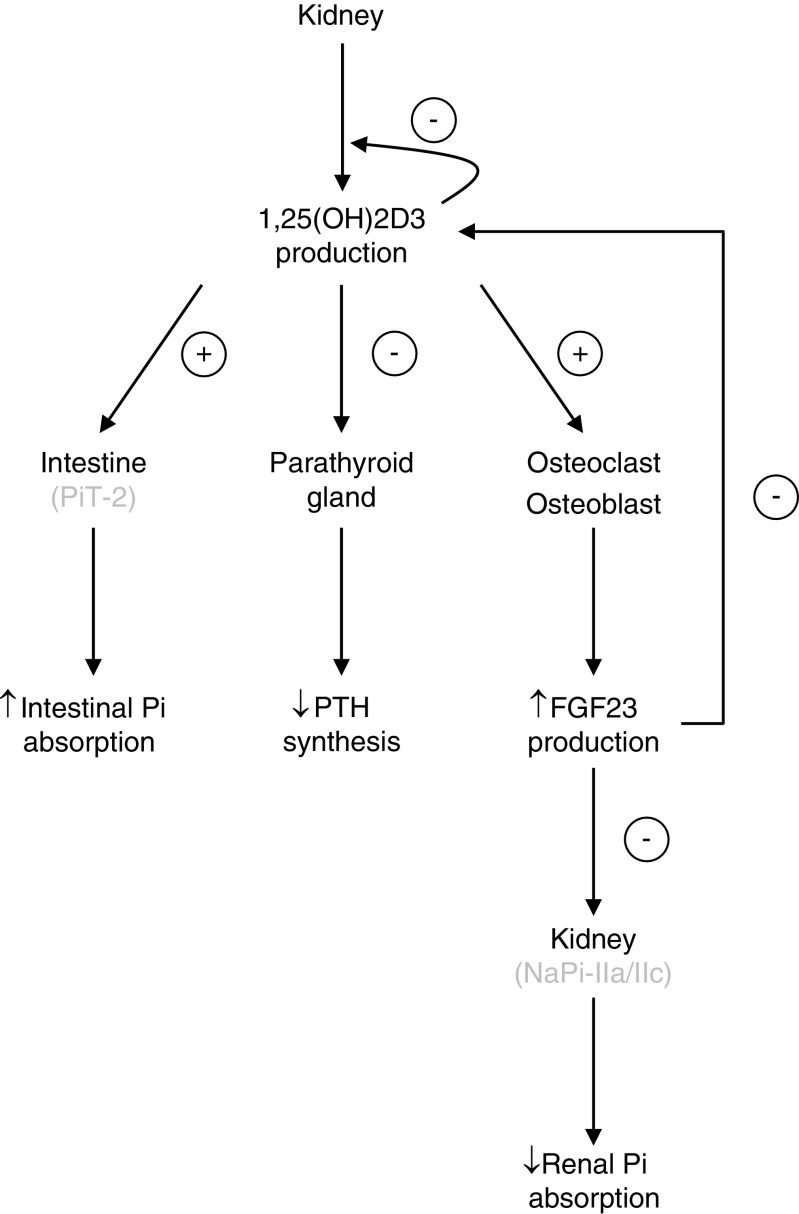
Regulation of Pi balance by vitamin D

It was originally thought that the parathyroid gland is not itself a target for vitamin D (see Fig. 2). Vitamin D deficiency associated with hypocalcaemia due to a decrease in calcium absorption from the diet is known to result in increased PTH secretion from the parathyroid [137]. It has been shown that elevated PTH resulting from hypocalcaemia mediates the induction of CYP27B1, which in turn stimulates the synthesis of 1,25(OH)2D3 via the nuclear orphan receptor 4A2, also known as NURR1 [173]. The discovery of 1,25(OH)2D3 as the active metabolite of vitamin D, allowed testing of the effect of vitamin D on the parathyroid gland. Silver et al. showed in vitro that 1,25(OH)2D3 decreases PTH production in bovine parathyroid cells in primary culture at the level of the transcription of the PTH gene [133]. They then confirmed in vivo the physiological relevance of these observations by administrating physiological doses to rats. They observed that 1,25(OH)2D3 decreases the level of PTH mRNA in the parathyroid gland of normal rats without changing the level of serum calcium. Moreover, 1,25(OH)2D3 receptor mRNA is highly expressed in the parathyroid gland, similar to what has been reported in the duodenum, and its abundance is amplified with 1,25(OH)2D3 administration, thereby increasing the effect of 1,25(OH)2D3 on PTH transcription [101]. Confirmation of the effect of 1,25(OH)2D3 on PTH levels in humans came from findings in patients with chronic kidney disease (CKD) [134] (see later).

As well as the direct effect of 1,25(OH)2D3 on PTH transcription, it was also shown that it can affect PTH concentrations via serum calcium and the calcium sensing receptor (CaSR). It has been reported that 1,25(OH)2D3 upregulates the transcription of the gene encoding the CaSR in the parathyroid gland, making it more sensitive to the ambient serum calcium concentration that can cause a decrease in PTH secretion [25].

Although it is well known that 1,25(OH)2D3 can regulate the metabolism and homeostasis of calcium and Pi, there is still a debate about direct effects of 1,25(OH)2D3 on Pi reabsorption in the PT. In normal rats, supplementation with 1,25(OH)2D3 decreases PTH secretion and Pi excretion, whereas in thyroparathyroidectomised (TPTX) rats, chronic administration of 1,25(OH)2D3 was reported to inhibit Pi reabsorption in the PT [28, 95]. The explanation may come from data suggesting an indirect action of 1,25(OH)2D3 on renal Pi handling via altered serum levels of a phosphatonin [135], one of the candidates being FGF-23 [110, 128]. Despite the fact that a 1,25(OH)2D3 responsive element has been identified in the promoter region of the human NaPi-lla gene [141], no clear evidence of a direct effect of 1,25(OH)2D3 has been reported. Moreover, in vitamin D receptor (VDR) or 1α-hydroxylase null mice, despite the fact that the expression of the NaPi-lla protein in vesicles prepared from apical BBM of the PT is significantly decreased compared with wild-type mice [123], no difference was observed in in the knockout mice after they were fed a low-Pi diet [27], which suggests that at least in mice the regulation of NaPi-lla abundance by low dietary intake of Pi may not be 1,25(OH)2D3-VDR-dependent.

### Parathyroid hormone

#### Effect of Pi and Ca^2+^ on PTH secretion

PTH is the major modulator of bone and mineral metabolism through its regulation of calcium and Pi homeostasis. Synthesis and cleavage of PTH occur within the parathyroid gland. PTH is a polypeptide synthesised in the endoplasmic reticulum following two successive cleavages: 115 amino acid pre-pro-PTH cleaved to 90 amino acid pro-PTH. Pro-PTH is then cleaved again to form an active mature full-length 84 amino acid PTH, which is stored in secretory granules within the parathyroid gland. The whole process of PTH synthesis, its cleavage and storage, is fast and has been estimated to take less than an hour. Active PTH is secreted when storage granules fuse with the outer membrane releasing the hormone into the extracellular compartment. In vitro, it has been shown that this mechanism is quick and regulated by the extracellular calcium concentration [Ca^2+^]_e_ [97]: an increase in extracellular [Ca^2+^]_e_ from 0.5 to 2.0 mM inducing a 50% reduction in PTH secretion. After release, PTH is rapidly removed from the serum by the kidney and the liver. Because the amount of active mature PTH is limited and its degradation rapid, most of the regulation of PTH is at the gene expression level [26].

As with synthesis, the secretion of PTH is also mainly under the control of [Ca^2+^]_e_. However, it can also be affected by an increase in serum Pi levels (upregulation), an increase in serum 1,25(OH)2D levels (downregulation) and potentially by an increase in FGF23 levels. [Ca^2+^]_e_ and Pi play an important role in the regulation of the abundance of PTH mRNA. In rats fed a diet high in Pi or low in calcium, decreased calcium and increased Pi serum concentrations were observed, and associated with an increase in PTH mRNA concentration [77, 92, 167]; although, PTH regulation by serum calcium or Pi occurs at the post-transcriptional level [77, 92]. Moallem et al. have performed nuclear transcript run-ons and shown that despite similar transcription rates in rats fed a normal or low calcium diet, there was a tenfold increase in PTH mRNA levels in hypocalcaemic animals, confirming that the effect of a low serum calcium is at the level of mRNA stability [92]. Kilav et al. have shown a similar post-transcriptional effect of a low serum Pi in hypophosphataemic rats, leading to a decrease in PTH mRNA concentration [77]. The role of protein-RNA interactions after transcription was also assessed in response to diet-induced hypocalcaemia and hypophosphataemia. It was shown that in the parathyroid gland of hypocalcaemic rats, protective cytosolic proteins bind to a defined region in the 3′-untranslated part of PTH mRNA (3′-UTR) that can prevent ribonucleases from degrading it; conversely, in hypophosphataemic animals, reduced binding was observed [92]. Serum calcium and Pi are important regulators of these cytosolic factors, helping to maintain the normal balance between degradation and protection. Using different PTH cDNA constructs, a 60 nucleotide sequence of the 3′-UTR has been identified as being the binding site for these parathyroid cytosolic proteins [92].

By using an in vitro assay, it has been shown that degradation of PTH mRNA is increased by 80% within 5 min when incubated with proteins from hypophosphataemic rats; whereas when PTH mRNA is incubated with proteins from control animals, it remains intact after 40 min. Furthermore, cytosolic proteins from hypocalcaemic rats increase the stability of PTH mRNA for up to 180 min, whereas the mRNA transcript without the 3′-UTR was not affected at all by cytosolic proteins from normal and low Pi rats [92]. Besides the degradation of mRNA, there are proteins such as AUF1 (A + U-rich element binding factor 1) that bind to the PTH mRNA to stabilise it [100, 102]. It is believed that the mRNA half-life is determined by a balance of these degrading and stabilising proteins, and that both serum calcium and Pi determine PTH mRNA levels by regulating the binding of these proteins to the 3′-UTR of the PTH mRNA.

#### Effect of PTH on renal pi transport

Pi homeostasis is maintained primarily by control of Pi excretion in the urine. Of the many mechanisms that regulate the urinary excretion of Pi, the effect of PTH is considered to be of major importance. In 1971, Agus et al. showed an inhibitory effect of PTH on Pi transport mediated by cAMP, the only known second messenger at the time [2]. Pi transport was also shown to correlate with NaPi-IIa transporter protein abundance, which was decreased by PTH [58]. In 2002, it was demonstrated that NaPi-IIa bound to apical membrane scaffolding PDZ domain-containing proteins such as NHERF-1. NHERF-1 null mice were found to be hypophosphataemic and that this was due to renal Pi wasting [57, 127].

NHERF-1 is bound to NaPi-IIa at the apical membrane and its role is to interact with the transporter, extending its time for residence and expression at the BBM [159]. PTH fails to inhibit Pi transport in NHERF-1 null mice, indicating an interaction between the PTH1^1^ receptor and NHERF-1 [33]. In PT cells, the PTH1 receptor is expressed at both apical and basolateral membranes [69] and signals via protein kinase C (PKC) and protein kinase A (PKA), respectively [152]. It has been shown that in the apical membrane, NaPi-IIa is associated with structural and anchoring proteins that play a role in regulating PTH signalling and functional responses [51, 74, 75]. In contrast, NHERF-2, which is also present at the PT in both humans and animals, does not seem to play a role in renal Pi handling [158, 160]. In animals, in the absence of NHERF-2, the serum concentration of Pi, the urinary excretion of Pi and abundance of NaPi-IIa in the PT were no different from those in wild-type control mice [34].

**Fig. 3:**
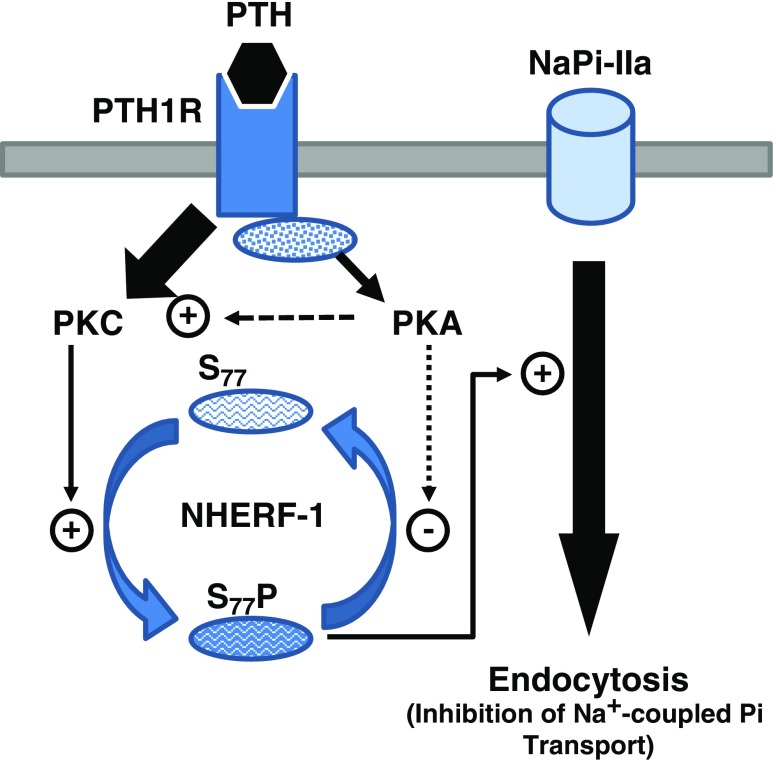
Downregulation of NaPi-IIa by PTH in renal proximal tubule (PT)cells (Adapted/modified from Cunningham et al. [32])

The regulation of Pi transport and NaPi-IIa surface expression in response to PTH occurs through the major second-message signalling pathways PKC and PKA used by the PTH1 receptor [35]. In the presence of the PKC inhibitor chelerythrine, PTH-mediated inhibition of renal Pi transport is completely abolished; whereas the PKA inhibitor Rp-cAMP has no effect, suggesting that PKC is key to PTH signal transduction, which is consistent with findings that PKC directly phosphorylates NHERF-1 [32] (see Fig. 3).

Pi transporters are not directly affected by PTH-activated second messengers, since no phosphorylation of the NaPi transporters has been reported [39]. In contrast, NHERF-1 has been reported in vitro to be directly phosphorylated by PKC, but not PKA, at a serine77 residue [159, 161, 162]. In vitro, it has been observed that PTH inhibition of Pi transport in PT requires the dissociation of the NaPi-IIa/NHERF-1 complex, which occurs following phosphorylation of serine77 of NHERF-1, causing a decrease in its affinity for NaPi-IIa [159]. Furthermore, it has been reported that phosphorylation of the threonine95 residue of the PDZ I domain of NHERF-1 by PTH-activated pathways is a necessary modification for PKC to phosphorylate the serine77 residue [163]. The decrease in affinity of the phosphorylated NHERF-1 for NaPi-IIa may facilitate its binding to other proteins such as myosin IV, resulting in the retrieval of the transporter from the BBM [19]. Using specific markers of different endocytic pathways and compartments (insulin for receptor-mediated endocytosis, horseradish peroxidase and FITC-dextran for fluid phase endocytosis, early endosomal antigen 1 for early endosomes and Igp120 for late endosomes/lysosomes), Bacic et al. have shown that after acute PTH administration in mice, NaPi-IIa is removed from the BBM by receptor-mediated endocytosis via clathrin-coated vesicles, early and late endosomes and degradation in lysosomes [9]. However, the direct participation of megalin, which serves as a receptor for uptake and endocytosis of multiple ligands in the PT, has not been established with certainty. Nevertheless, it has been observed that NaPi-IIa and megalin are colocalised at the BBM [7, 8], but then follow two distinct routes after PTH-induced internalisation, with megalin recycled to the apical membrane [30] and NaPi-IIa degraded in lysosomes [111, 151].

## Non-hormonal factors that affect Pi balance

### Effects of dietary Pi on the parathyroid gland

For many decades, the effect of Pi on parathyroid cell function (see “Effect of Pi and Ca^2+^ on PTH secretion” section for more details) has always been considered secondary to a decrease in serum calcium and 1,25(OH)2D3 levels [132]. However, it has been demonstrated in CKD patients, as well as in animal models, that the correction of serum Pi alone, with no change in serum calcium or 1,25(OH)2D3 levels, is able to correct serum PTH levels by regulating PTH mRNA [4, 103, 132]. This regulatory mechanism seems to involve the inhibition of cytosolic phospholipase A2 (cPLA2) [3]. Moreover, it has been reported that PiT-1 is the only NaPi cotransporter in the parathyroid grand [143]. These authors have shown that the abundance of PiT-1 mRNA in the parathyroid gland varies according to the intake of Pi. In rats fed a low-Pi diet, the abundance of Pit1 mRNA is higher than when on a high-Pi diet. Tatsumi and al. proposed that PiT-1 present in the parathyroid gland may be involved in the effects of Pi and vitamin D on parathyroid function [143].

### Effects of dietary Pi on NaPi transporters

Dietary Pi is an important regulator of renal Pi reabsorption. It is well known that dietary Pi restriction is associated with an increase in PT Pi reabsorption. Variations in serum Pi caused by changes in dietary Pi intake are paralleled by plasma PTH levels: high Pi intake is associated with higher PTH levels and a low Pi diet is associated with reduced PTH levels [22]. However, studies in thyroparathyroidectomised rats have shown that adaptation to chronic Pi restriction remains intact, demonstrating that PTH is not the major regulator in this setting [138]. It has been shown that mice fed with a low Pi diet exhibit an elevated abundance of NaPi-IIa mRNA and protein; both transcriptional and post-transcriptional levels of NaPi-IIa seem to be affected by a change in Pi concentration in the diet [76, 93, 94]. Kido at al. have identified in cortical renal nuclear extracts isolated from mice fed a low Pi diet a DNA sequence responsible for the Pi response, which they named the Pi Responsive Element (PRE). The PRE of the NaPi-IIa gene promoter has a region with 9 of 10 bp identity to the binding element of the yeast Pi-responsive transcription factor Pho4. At the centre of this region, there is a CACGTG motif, the core recognition site for the helix-loop-helix family of transcription factors [76]. The 5-CACGTG-3 motif is sufficient to confer transactivation by dietary Pi deprivation. Using a yeast one-hybrid system, the authors isolated the transcription factor TFE3 that can bind to the 5-CACGTG-3 motif of PRE of NaPi-IIa, which is sufficient to confer transactivation by dietary Pi deprivation. During Pi diet depletion, a significant increase in the amount of TFE3 mRNA in the kidney is observed, suggesting a role in transcriptional regulation of the NaPi-IIa gene by dietary Pi [76]. Under hypophosphataemic conditions, the stability of NaPI-IIa mRNA is also increased. Moz et al. have identified the mechanisms involved in the post-transcriptional effect of dietary Pi. They have shown that a protein-RNA interaction with the 5-UTR region of NaPi-IIa occurs, increasing its translation [93, 94]. The stabilisation of NaPi-IIa by renal cytosolic proteins of rats fed a low Pi diet depends on the presence of a region within the 3 terminal 698 bp of the mRNA. Using an in vitro degradation assay, they observed that the protein-binding region of NaPi-IIa mRNA functions as a cis-acting stability element and that this binding is increased during hypophosphataemia, increasing the stabilisation of NaPi-IIa mRNA [93, 94]. Similar to NaPi-IIa, the expression of NaPi-IIc is significantly increased in the PT apical membrane of rats fed a chronically low Pi diet [106]. However, any direct interaction of Pi with NaPi-IIc activity and protein abundance has not been defined.

### Direct Pi sensing

Direct Pi sensing has been suggested as another mechanism by which Pi transport can be regulated in the renal PT. Activation of MAPK by calcium-Pi crystals in primary fibroblasts was described by Nair et al. [99] and Beck et al. have shown that 5 to 10 mM Pi is sufficient to activate MAPK in MC3T3 mouse fibroblast cells [13]; several more studies have demonstrated an activation of MAPK by inorganic Pi in various cell lines, including human embryonic kidney (HEK) 293 cells [168]. In the latter cells, extracellular Pi activates Raf/MEK/ERK pathway via FGF receptors (FGFR1). Since FGFR1 is also activated by FGF23, which increases renal Pi excretion, it is likely that by activating FGFR1, extracellular Pi can stimulate the urinary excretion of Pi. However, it is still unknown whether physiological concentrations of extracellular Pi can interact with MAPK signalling in vivo.

## Disorders Pi balance

Disorders of Pi homeostasis can occur in a range of clinical conditions. The three main causes of disturbed Pi balance are changes in oral intake, changes in gastrointestinal tract reabsorption and changes in renal excretion. Since serum inorganic Pi accounts for only a small fraction of total body Pi, alterations in serum Pi levels can occur when total body Pi is low, normal or high.

### Hyperphosphataemia

#### Chronic kidney disease

Hyperphosphataemia (see Table 1) is a major cause of morbidity and mortality in patients with CKD [73] and can also be a cause of acute kidney injury (AKI). Apart from cell shifts, which are rare, and tend to occur in acidotic and hypoxic states, or with decreased intracellular consumption and increased cellular release of Pi, hyperphosphataemia occurs more commonly in the presence of renal insufficiency and is due to a decrease in Pi excretion from reduced filtration, despite an associated decrease in PT Pi reabsorption [59, 82]. CKD associated with a glomerular filtration rate < 30 mL/min is usually associated with an increase in serum Pi [82]. In human as well as in animals, calcium and Pi disturbances are a hallmark of CKD and are implicated in the development of vascular and valvular calcification, microvascular disease and endothelial dysfunction [115, 121].

**Table 1:** Genetically determined hyperphosphataemic disorders with their phenotype Online Mendelian Inheritance in Man (OMIM) numbers; the genes involved together with their MIM numbers and the chromosomal locations of the genes

Disorders	Abbreviation	Phenotype OMIM number	Inheritance	Gene/locus	Gene/locus MIM number	Gene location
*Hyperphosphataemia*						
Hyperphosphatemic familial tumoral calcinosis type 1	HFTC1	211900	Autosomal recessive	GALNT3	601756	2q24.3
Hyperostosis-hyperphosphataemia syndrome	HSS	610233	Autosomal recessive			
Hyperphosphatemic familial tumoral calcinosis type 2	HFTC2	617993	Autosomal recessive	FGF23	605380	12p13.32
Hyperphosphatemic familial tumoral calcinosis type 3	HFTC3	617994	Autosomal recessive	KL	604824	13q13.1
Pseudohypoparathyroidism type Ia	PHP1A	103580	Autosomal dominant	GNAS	139320	20q13.32
Pseudohypoparathyroidism type Ib	PHP1B	603233	Autosomal dominant	STX16 GNASAS1 GNAS	603666 610540 139320	20q13.32
Pseudohypoparathyroidism type Ic	PHP1C	612462	Autosomal dominant	GNAS	139320	20q13.32
Pseudohypoparathyroidism type II	PHP2					
Familial isolated hypoparathyroidism	FIH	146200	Autosomal dominant	GCM2	603716	6p24.2
Hypoparathyroidism			Autosomal dominant or Autosomal recessive	PTH	168450	11p15.3
Blomstrand chondrodysplasia	BOCD	215045	Autosomal recessive	PTHR1	168468	3p21.31

Both CKD patients and experimental animal models of kidney failure show increased serum PTH and FGF23 levels. In humans, increased FGF23 levels appear before a detectable rise in PTH and hyperphosphataemia, suggesting that an increase in FGF23 may be the earliest sign of disordered mineral metabolism in CKD [64]. In 4-week adenine-enriched diet (ADE)-fed mice, elevation of serum Pi, PTH and FGF23 is accompanied by the clinical features of CKD such as elevated urea and creatinine, and elevated renal expression of tubular injury markers like NGAL, whereas in partial nephrectomised (5/6 Nx) mice and in 2-week ADE-treated mice, despite the clinical features of renal failure, the blood Pi levels were normal [115]. In the renal tubule, a low expression of transmembrane-α-klotho is generally associated with kidney tubular cell resistance to FGF23 leading to hyperphosphatemia. Soluble α-klotho, which is thought to mimic the expression of transmembrane α-klotho seems to be another early marker of renal failure since a decrease in circulating soluble α-klotho has been observed during the very early stage of CKD [117]. This phenotype is also observed in 2- and 4-week ADE-treated mice and in a mouse model with partial deletion of klotho in distal tubular segments (Ksp-KL^−/−^), which is associated with hyperphosphataemia and elevated FGF23 [107, 115]. Furthermore, acute and chronic inflammation are also recognised to be stimuli for elevated FGF23 in CKD patients, as well as in normal mice [38, 115]. Growing evidence for a link between Pi and FGF23 excess and increased cardiovascular disease risk in CKD has led to a recent focus on Pi- and FGF23-lowering therapies. Based on the use of dietary restriction and Pi binders, different studies have shown a decrease in serum Pi and FGF23 but have so far failed to prevent progression of vascular calcification [20].

### Other causes

Hyperphosphataemia as a result of increased PT reabsorption of Pi occurs in hypoparathyroidism, acromegaly, treatment with bisphosphonates and following vitamin D toxicity, with hypercalcaemia also reducing Pi excretion and PTH secretion [53, 108, 131, 154]. In autosomal recessive familial tumoral calcinosis, renal Pi reabsorption is increased, often associated with raised levels of 1,25(OH)2D3. Originally attributed to mutations in *GALNT3*, a glycosyltransferase involved in FGF23 breakdown, mutations have also been found in FGF23 itself and klotho [43]. This condition is the mirror image of X-linked hypophosphataemic rickets in which increased FGF23 reduces Pi reabsorption and increases urinary Pi excretion [63]. Other clinical scenarios of Pi overload in which hyperphosphataemia can occur are following an acute exogenous Pi load (e.g., Pi-containing laxatives), in tumour lysis syndrome and in rhabdomyolysis [5, 45, 86].

Pseudohypoparathyroidism (PHP) types Ia, Ib and II are autosomal dominant, sporadic/autosomal dominant, and sporadic disorders, respectively, and conditions associated primarily with resistance to the parathyroid hormone in the renal PT [12]. They are associated with hypocalcaemia, hyperphosphataemia, and elevated serum concentration of PTH, which reflect end-organ resistance to PTH [164]. In type Ia (PHP-Ia), end-organ resistance is usually not limited to the actions of PTH but can also affect other hormones such as thyrotropin and gonadotropins [164]. Patients affected by PHP-Ia also have the physical features of Albright hereditary osteodystrophy (AHO), including obesity, short stature, brachydactyly and ectopic tissue ossification. PHP-Ia is associated with inactivating defects of the stimulatory G protein α subunit (Gsα) caused by heterozygous mutations in one of the 13 exons of GNAS that encode Gsα [78]. Type Ib is another variant in which end-organ resistance appears to be limited primarily to the actions of PTH in the renal cortex. Unlike PHP-Ia, the mutation responsible for PHP-Ib is in a cis-acting element of GNAS that controls exon A/B methylation. The loss of methylation at this site leads to a profound reduction or a complete lack of Gsα expression in the renal PT (and possibly a few other tissues) [169]. Type II is a third variant of PHP in which patients do not show AHO, despite an impaired phosphaturic response. It is interesting to note that in PHP-II, patients show an increased renal Pi excretion associated with normal urinary excretion of cAMP; whereas patients with PHP-I fail to show increases in urinary excretion of either cAMP or Pi [47, 126].

### Hypophosphataemia

Clinical hypophosphataemia (see Table 2) is typically manifest as a serum Pi < 0.32 mM [48]. Chronic hypophosphataemia is usually due to diminished Pi reabsorption associated with an increase in circulating PTH levels (primary or secondary hyperparathyroidism), vitamin D deficiency or resistance [48, 139]. Hypophosphataemia is often mild in primary hyperparathyroidism, but can be more severe when secondary to vitamin D deficiency in which gastrointestinal Pi absorption is also reduced. Cell shifts can reduce serum Pi levels during refeeding in malnourished individuals, especially alcoholics, following treatment of diabetic ketoacidosis, and during parenteral feeding.

**Table 2:** Genetically determined hypophosphataemic disorders with their phenotype Online Mendelian Inheritance in Man (OMIM) numbers; the genes involved together with their MIN numbers and the chromosomal locations of the genes

Disorders	Abbreviation	Phenotype OMIM number	Inheritance	Gene/locus	Gene/locus MIM number	Gene location
*Hypophosphataemia*						
X-linked hypophosphataemia	XLH	307800	X-linked dominant	PHEX		Xp22.11
Autosomal dominant hyphosphatemic rickets	ADHR	193100	Autosomal dominant	FGF23	605380	12p13.32
Autosomal dominant hyphosphatemic rickets	ADHR1 or ARHP	241520	Autosomal recessive	DMP1	600980	4q22.1
Autosomal dominant hyphosphatemic rickets	ADHR2	613312	Autosomal recessive	ENPP1	173335	6q23.2
Hereditary hypophosphatemic rickets with hypercalciuria	HHRH	241530	Autosomal recessive	SLC34A3	609826	9q34.3
Vitamin D-resistant rickets type 1A	VDDR1A	264700	Autosomal recessive	CYP27B1	609506	12q14.1
Vitamin D-resistant rickets type 2A	VDDR2A	277440	Autosomal recessive	VDR	601769	12q13.11
Familial hypocalciuric hypercalcemia type I	HHC1	145980	Autosomal dominant	CASR	601199	3q13.3-q21.1
Neonatal severe hyperparathyroidism	NSPH	239200	Autosomal recessive	CASR	601199	3q13.3-q21.1
Jansen type of metaphyseal chondrodysplasia		156400	Autosomal dominant	PTHR1	168468	3p21.31
Hypophosphataemica nephtolithiasis/osteoporosis-1	NPHLOP1	612286	Autosomal dominant	SLC34A1	182309	5q35.3
Hypophosphataemica nephtolithiasis/osteoporosis-2	NPHLOP2	612287	Autosomal dominant	SLC9A3R1	604990	17q25.1
Osteoglophonic dysplasia	OGD	166250	Autosomal dominant	FGFR1	136350	8p11.23
Opsismodysplasia	OPSMD	258480	Autosomal recessive	INPPL1	600829	11q13.4
Schimmelpenning-Feuerstein-Mims syndrome, somatic mosaic	SFM	163200	Postzygotic somatic mutation	NRAS	164790	1p13.2
				HRAS	190020	11p15.5
				KRAS	190070	12p12.1
Mc Cune-Albright fibrous dysplasia, somatic mosaic	MAS/FD	174800	Postzygotic somatic mutation	GNAS	139320	20q13.32
Neurofibromatosis type I	NF1	162200	Autosomal dominant	NF1	613113	17q11.2
Neurofibromatosis type II	NF2	101000	Autosomal dominant	NF2	607379	22q12.2

Oncogenic osteomalacia is associated with renal phosphate wasting leading to hypophosphataemia, osteomalacia and abnormal vitamin D metabolism [62]. The first symptoms of oncogenic osteomalacia are typically fatigue, muscle weakness, bone pain, fractures and osteomalacia [29]. Causes are thought to be due to production and secretion of proteins—phosphatonins—by mesenchymal tumours, of which FGF23, sFRP4 and FGF-7 are those identified so far (see article *Physiological regulation of phosphate: klotho, FGF23* for full review). Surgical removal of the tumour can reverse the symptoms and normalise levels of serum Pi and 1,25(OH)2D3. Non-surgical treatment consists of phosphate supplements and vitamin D (calcitriol) [37].

Autosomal dominant hypophosphataemic rickets (ADHR) presents as rickets, hypophosphataemia, hyperphosphaturia, fatigue, bone pain, and bone deformities with inappropriately low or normal vitamin D3 levels. ADHR is the prototype disorder of primary FGF23 excess [72]. A mutation in *FGF23* has been described in its cleavage motif, resulting in increased levels of active FGF23 [72]; this finding has been confirmed in mice transgenic for proteolytically resistant human FGF23 [11].

X-linked hypophosphatemic rickets (XLH), mentioned earlier, is the most common form of hereditary rickets and is due to a loss of function mutation in the phosphate regulating gene with homology to endopeptidases on the X chromosome (PHEX) gene. XLH is characterised by hypophosphataemia due to renal phosphate wasting, leading to rickets, inappropriately normal to low concentrations of 1,25(OH)2D3 and high circulating levels of FGF23 [42]. Short stature and rachitic osseous lesions are characteristic features of XLH, although the severity of these manifestations is highly variable among patients [42]. Recently, a new nonsense mutation (p.E145*) in exon 4 of PHEX has been predicted to be responsible for XLH [83]. Animal models of XLH have demonstrated a defect in PT phosphate reabsorption and decreased expression of NaPi-IIa and NaPi-IIc [144–146]. In mice, FGF23 levels are high and it has been suggested that PHEX and FGF23 may regulate each other’s expression, the loss of PHEX leading to higher expression levels for FGF23 [84, 109]. However, the interplay between PHEX and FGF23 is still not fully understood.

## Defects in Na^+^-coupled Pi cotransporters

### NaPI-IIa (SLC34A1) mutations

Impaired NaPi-IIa function is associated with a variety of overlapping clinical syndromes that include hypophosphataemia, nephrolithiasis, osteoporosis, renal Fanconi syndrome and CKD. An early analysis of 20 patients with urolithiasis or bone demineralisation and persistent idiopathic hypophosphataemia associated with a decrease in maximal renal Pi reabsorption was published in 2002. Two patients, one with urolithiasis and one with bone demineralisation, were shown to have two distinct mutations on one allele of the gene encoding NaPi-IIa. The expression of the two mutants in oocytes showed reduced Pi-induced current and Na^+^-coupled Pi uptake. However, these gene mutations were also found in several subjects with normal renal Pi excretion, suggesting that their relevance to renal Pi handling required more clarification [114]. In 2010, the sequence analysis of NaPi-IIa in two siblings with autosomal recessive proximal tubulopathy associated with severe renal Pi wasting, hypophosphataemic rickets and renal failure revealed a 21-nucleotide stretch of duplicated sequence. Functional and expression studies of the mutant gene product in oocytes and opossum kidney cells showed a complete loss of function of the mutant NaPi-IIa as a consequence of its mislocalisation within the intracellular compartment and failure to reach the cell membrane [89]. More recently, whole-exome sequencing in two unrelated patients with idiopathic infantile hypercalcaemia with partial proximal tubulopathy revealed a homozygous loss-of-function inserted duplication (p.I154_V160dup) in NaPi-IIa. The in vitro localisation and trafficking analysis of p.I154_V160dup mutant indicated aberrant retention at the endoplasmic reticulum in an immature and under-glycosylated state, leading to premature proteasomal degradation of NaPi-IIa [40]. Homozygous mutations in NaPi-IIa seem to be responsible for renal Pi wasting, although more studies are needed to provide stronger evidence for their biological and clinical importance in human kidney function.

### NaPI-IIc (SLC34A3) mutations

Hereditary hypophosphataemic rickets with hypercalciuria (HHRH) is a very rare disease caused by biallelic mutations in NaPi-IIc [16, 61, 87]. First described in 1985 by Tieder [149], this inherited disease is characterised by decreased renal Pi reabsorption, hypophosphataemia, vitamin D3 refractory rickets, hyperphosphaturia, hypercalciuria, elevated circulating 1, 25(OH)2D3 levels, and low serum parathyroid hormone (PTH) levels, leading to growth retardation, limb deformities, bone pain, muscle weakness, rickets and osteomalacia [16, 87, 149, 150]. Unlike mice, NaPi-IIc seems to have a more important role in calcium homeostasis: kidney-specific deletion of NaPi-IIc in mice does not affect renal calcium or Pi handling [98, 125] .

In conclusion, phosphate homeostasis is the result of a highly complex metabolic, hormonal, ion transporter and whole organ interplay that serves to maintain serum Pi levels within narrow limits. Both Pi deficiency and overload are reflected in serum Pi levels and have their consequences for disease, particularly the emergence of Pi as an important risk factor for cardiovascular disease and progression in CKD.

## Footnotes

1 PTH1 receptor binds PTH and PTH-related peptide or hormone, whereas PTH2 receptor has limited tissue distribution (mainly brain and pancreas) and is selective for PTH.
